# Methylation array signals are predictive of chronological age without bisulfite conversion

**DOI:** 10.1007/s11357-025-01785-5

**Published:** 2025-07-29

**Authors:** Hunter L. Porter, Victor A. Ansere, Ram Babu Undi, Walker Hoolehan, Cory B. Giles, Chase A. Brown, David Stanford, Mark M. Huycke, Willard M. Freeman, Jonathan D. Wren

**Affiliations:** 1 Oklahoma Medical Research Foundation, Oklahoma City, OK 73104-5005 USA; 2 University of Oklahoma Health Sciences Center, Oklahoma City, OK USA; 3 Oklahoma Nathan Shock Center, Oklahoma City, OK 73,104 USA; 4 Oklahoma City Veterans Affairs Medical Center, Oklahoma City, OK 73,104–5005 USA

**Keywords:** Age-predictive, Bisulfite, Methylation

## Abstract

DNA methylation data has been used to make “epigenetic clocks” which attempt to measure chronological and biological aging. These models rely on data derived from bisulfite-based measurements, which exploit a semi-selective deamination and a genomic reference to determine methylation states. We found that non-methylation factors lead to “pseudomethylation” signals that are both confounding of epigenetic clocks and uniquely age predictive. We also failed to predict age using human genotyping arrays, but found that epigenetic clocks were overrepresented near genomic regions whose methylation state is dependent upon sequence variants. Quantifying these covariates in aging studies will be critical to building better clocks and designing appropriate studies of epigenetic aging.

**Supplementary Information:**

The online version contains supplementary material available at 10.1007/s11357-025-01785-5.

## Introduction

Epigenetic alterations and genomic instability are each hallmarks of the aging process [1]. Prior studies have shown that high-throughput epigenetic DNA modification assays, such as DNA methylation arrays, can be used to make “epigenetic clock” models that are predictive of chronological age [2, 3]. Further studies on DNA methylation in aging led to the development of second-generation clocks purporting to measure “biological aging” [4, 5]. The relationship between predicted “accelerated aging” and age-related disease and dysfunction is unclear, and often contradictory [6, 7]. Nonetheless, clock studies suggest a molecular mechanism that might be a surrogate endpoint for evaluating anti-aging interventions in humans [8]. The use of epigenetic clocks is rapidly on the rise [4, 5, 9–12], and has even inspired new theories of aging [10]. Most methylation studies, however, rely on sodium bisulfite conversion to identify the methylation status of CpGs [13].

**Fig. 1:**
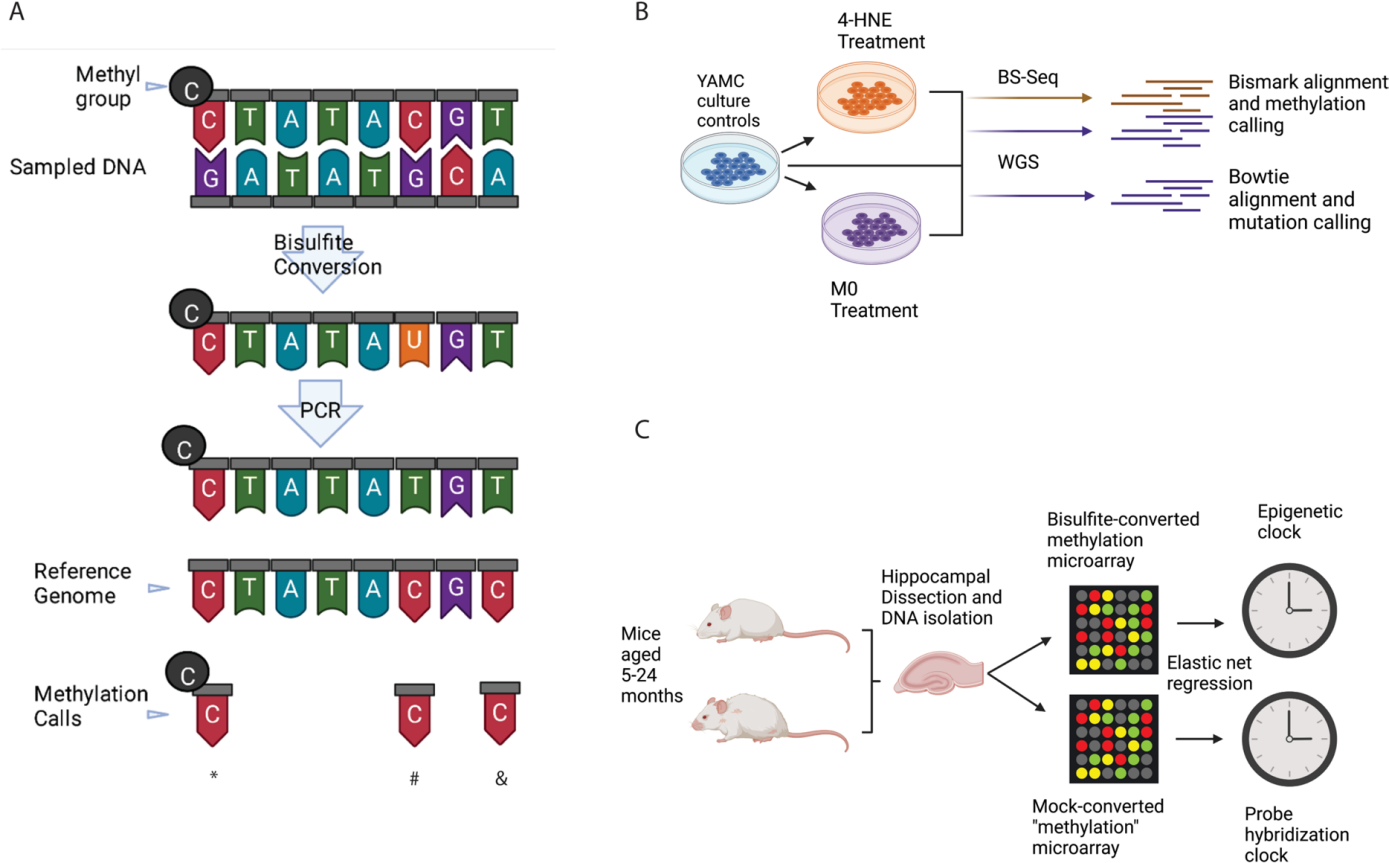
Conceptual framework and experimental methodology. **A** Diagram showing how bisulfite treatment is used to make methylation calls. Standard methylation calls (denoted by an asterisk) and unmethylated calls (number sign) are contrasted with an example of a C>T mutation being read as unmethylated (ampersand). **B** Experimental paradigm for paired sequencing experiments to demonstrate the relationship between methylation and mutations in vitro. **C** Experimental paradigm for generating converted and unconverted “methylation” data using methylation microarrays and subsequent epigenetic clock analysis

Sodium bisulfite catalyzes the deamination of cytosine to uracil [14]. This method assumes that thymines observed at what are cytosines in the reference genome (including microarray probes) are due to bisulfite conversion of unmodified cytosine (Fig. 1A). A potential confound is that accumulation of mutations with aging is well known [15]. More specifically, there is significant evidence that deamination mutations accumulate with aging [16] and may even be age predictive [17]. As well, germline mutations can be predicted from alterations to methylation array signals [18], but the somatic mutation signal does not follow allelic ratios and may be difficult or impossible to identify within the same data. Mutations in CpG dinucleotides are predictive of time in taxonomic contexts [11], and the mutation rate appears to be related to the methylation level of cytosines [19]. Cytosine to thymine (C>T) substitutions are the most abundant in the genome with aging [16, 20]. C>T mutations were previously linked to patient age in a study analyzing mutations in human cancers; they found two “clocks” in mutational signatures and proposed an etiology of cytosine deaminations correlated with patient age [21]. Despite the enrichment for age-related mutations in cytosines, the number of mutations confidently detected is lower than we expect to explain clocks entirely.

Thus, it should not be controversial to suggest part of the age-related signal from epigenetic clocks is due to mutations, but the degree and effects are unclear. If aging creates many somatic C>T mutations, they might induce an apparent “genomic hypomethylation” with aging, which was previously a commonly accepted theory [22]. However, T>C mutations are almost as abundant in aged samples and may create new methylatable loci [16]. The array data, where epigenetic clocks are most thoroughly studied in humans, further complicate the equation by hybridizing physical DNA molecules — allowing even other mutations to influence affinity [23]. Answering this is key to understanding the biological ramifications of epigenetic clocks, and the behavior we might expect if they are to be a biomarker of aging that is responsive to treatment [24].

In a previous report [25], we identified that (1) epigenetic clocks can be trained to use a variety of redundant loci to predict age; (2) epigenetic clocks use loci that exhibit age-related changes much smaller than we expect to be physiologically impactful; and (3) the age-predictive loci are enriched outside of coding regions of the genome and are somewhat associated with annotated eQTLs. These observations, and the previously described context in the literature, led us to question if age-predictive methylation changes could be explained by the accrual of mutations in regions with low selection pressure, such as in pseudogenes in taxonomic analyses [26]. The idea that mutations could affect both direct measurements at a given locus and alter the methylation status of other CpGs as a result [27, 28] could boost the signal from local mutations into a common change within a methylation probe, especially if clonally expanded. It is worth noting that the somatic mutation rate with aging is still low [16], but enrichment for CpG sites and the fact that methylation may affect the mutation rate [29] makes deconvolving the signal difficult.

To answer these questions, we first attempted to understand how somatic mutations relate to readouts of DNA methylation by paired whole-genome sequencing (i.e., untreated, to detect mutations) and bisulfite sequencing from mouse colon epithelial cell lines transformed into cancer cells by mutagenic exposures (Fig. 1B). Our first hypothesis was that somatic mutations would be enriched in regions of altered DNA methylation and, further, that mutations within unconverted sequencing data would be read as loss of methylation when analyzed using bisulfite analysis tools. Next, we tested if microarrays could measure signals other than DNA methylation and produce age-predictive clocks. To this end, we generated Illumina Mouse Methylation array data using bisulfite-converted DNA or DNA subjected to a mock conversion process lacking bisulfite (unconverted). These data were isolated from the hippocampi of mice between 5 and 24 months of age with a total *N* of 38. We then trained epigenetic clocks using converted and unconverted data (Fig. 1C). Our second hypothesis was that we could generate models with similar age predictions using paired converted and unconverted DNA signals from methylation arrays.

## Methods

### Cancer cell culture

Immortalized young adult mouse colon (YAMC) epithelial cells were assigned to control or treatment groups. Treated cells were transformed as previously described [30]. Briefly, cells were co-cultured with macrophages polarized by Enterococcus faecalis infection or exposed to trans-4-hydroxy-2-nonenal (4-HNE). Macrophage exposure occurs in a dual-chamber system for 72 h with 96 h of recovery, while 1 µM 4-HNE exposure is done in 1-h treatment windows with 1 week of recovery repeated over 16 treatments.

### Mouse sample collection

C57BL/6 mice were obtained from the National Institute on Aging (NIA) aging colony at 4–18 months of age. Some mice were aged additionally for collection, up to 24 months of age. Mice were sacrificed at 4, 5, 8, 11, 14, 18, and 24 months of age; then, hippocampi were harvested, snap frozen in liquid nitrogen, and stored at − 80 °C until used. All animal experiments were performed according to protocols approved by the OMRF Institutional Animal Care and Use Committee.

### Whole-genome shotgun sequencing

Whole-genome shotgun sequencing (WGSS) libraries were prepared using Swift Biosciences Turbo v2 library preparation kits as per the manufacturers’ protocol (Swift Biosciences, Ann Arbor, MI). Briefly, DNA from each respective sample was simultaneously fragmented, end-repaired, and treated to create a single A-base overhang on each end. Following an SPRI bead-based cleanup, the DNA fragments were ligated to a unique dual-indexed adapter set. The prepared libraries were fluorometrically quantified with a Qubit fluorometer (Life Technologies, Grand Island, NY) and pooled at equimolar concentrations. The pool was then precisely quantified via qPCR with a NEBNext Library Quant Kit for Illumina (New England Biolabs, Ipswich, MA) on a LightCycler 480 instrument (Roche Diagnostics, Indianapolis, IN). The pool was then sequenced on an Illumina NovaSeq 6000 instrument using an S4 flow cell and paired-end 150 bp reads.

### Bisulfite-capture sequencing

Bisulfite oligonucleotide capture sequencing (BOCS) was performed utilizing genomic DNA, which is made into a sequencing library followed by bisulfite conversion. After conversion, the target genomic regions of interest are captured with probes against methylated and unmethylated versions of the targeted genomic regions. The target regions are then captured and amplified prior to sequencing. Targeted regions of the mouse genome include 109 Mb containing most of the annotated promoters and CpG islands. In total, nearly 3 million CG and 28 million CH sites are in the targeted regions.

### Variant calling pipeline

We used the variant calling pipeline from Dan Bolser at EMBL-EBI [31] with a few modifications. Briefly, samples were aligned to mm10 using Bowtie2 with very-sensitive settings. The produced.sam files were converted to bams and sorted using samtools. Then, bcftools’ mpileup was used to generate vcf files and variants were called using bcftools call. For visualizing methylation and pseudomethylation over variants, loci present after coverage filtering in all samples and both modalities were intersected with variants from these variant calls.

### Methylation microarrays

Isolated hippocampal DNA samples were split and assigned to one of three groups: bisulfite-converted (converted), mock bisulfite treatment (unconverted), or an even more stringent “no touch” control (data not shown). Bisulfite-converted samples were treated using the Zymo EZ DNA Methylation Kit following the manufacturer protocol with minor modifications by Illumina [23]. Unconverted samples were treated identically without the addition of bisulfite reagent but with the additional handling time and heat exposure critical to bisulfite conversion. No touch samples were exposed to neither bisulfite reagent nor the heating cycles and are available in the GEO records even though they were not analyzed here. Sample DNA was then enzymatically fragmented and hybridized to the BeadChip and washed before extension, staining, and imaging. Imaging-generated.idat files were then parsed into matrices of methylation loci X sample using methylprep [32]. Missing values were imputed using sklearn’s KNN Imputer [33].

### Clock training and analysis

Epigenetic clocks were fit as previously described [25] using sklearn’s elastic net regression implementation (ElasticNetCV) with data split into 75% test and 25% train sets. For repeated clock trainings, the random state and train/test split were altered between each replicate, with 100 models being fit for each data type and data subset. Training was done using two-fold cross-validation and maxiter = 5000–15,000. Primary feature selection was done using sklearn’s mutual information regression with the target of chronological age in months, excluding sites with computed MI < 0.20 in the training split. Over/underrepresentation of loci in clock models was computed using the hypergeometric test implemented in statsmodels [34]. Comparisons of clock accuracy and error were performed using scipy stats’ one-way ANOVA and Tukey’s post hoc test implementations. This same approach was used to train human genotyping clocks from Gagliano et al. [27], using both raw signal and allele calls. Age labels were obtained from our label extraction model [35]. Model scores were evaluated using sklearn’s score and mean absolute error functions, with some models returning scores arbitrarily worse than the mean. Comparison of converted and unconverted clock age acceleration was performed using scipy stats’ pearsonr() implementation.

### Methylation/pseudomethylation analysis

WGSS fastq’s were aligned to the mm10 reference genome using Bowtie 2 [36] or Bismark [37] with sensitive parameters (*N* = 1, *L* = 20). Bismark’s methylation extractor was used to make methylation calls from both bisulfite sequencing data (deamination by bisulfite) and whole-genome sequencing data (deamination by mutation). Loci were then filtered to coverage > 10 for all samples across both data types, and further intersected against independently called variants as described above.

### Over/underrepresentation testing

Comparing the distribution of our identified mouse clock sites with respect to genomic features, as well as human clock sites with ASM-QTLs, was performed using hypergeometric tests implemented in statsmodels [38] to compare overlapping features against their respective backgrounds. The relevant clock sites were obtained directly from the manuscripts for each clock [3, 4, 39], and backgrounds were defined as all sites that could be used for training the models. The DunedinPACE background was the smaller normalization set rather than all array loci. To facilitate a common analysis, Horvath’s original clock sites and their background were lifted forward to hg19. The ASM-QTLs and their affected CpGs were lifted backward to hg19 from GRCh38. Tables for each genomic feature were obtained for the mm10 genome build from UCSC table browser in bed format. Intersections for each background and positive set of loci were performed using pybedtools [40] to generate the contingency tables for hypergeometric tests to determine the odds ratios and confidence intervals.

### Use of research animals

The Institutional Animal Care and Use Committee (IACUC) at the Oklahoma Medical Research Foundation approved all animal procedures, which were conducted following the guidelines outlined in the National Institutes of Health (NIH) Guide for the Care and Use of Laboratory Animals. The mice were kept at the Oklahoma Medical Research Foundation in a HEPA barrier environment under specific pathogen-free (SPF) conditions. Mice were euthanized by cervical dislocation, followed by rapid decapitation, in accordance with the AVMA Guidelines for Euthanasia of Animals.

### Use of AI

ChatGPT was used to help refactor code for presentation and figure generation. Code was primarily used for generating plots or reshaping data from one format to another for plotting. Most prompts consisted of pre-existing code with the request of refactoring into functions or reformatting data consistently across datasets.

### Data availability

Sequencing data are available on NCBI’s Sequence Read Archive (SRA) under PRJNA1182740. Mouse microarray data are available on NCBI’s Gene Expression Omnibus (GEO) under GSE281062.

## Results

### Mutations can be read as losses of DNA methylation in bisulfite sequencing data

Our initial experiments were designed to serve as proof of concept for somatic mutations affecting methylation readouts. We wanted to examine a condition with both apparent methylation changes and a high number of somatic mutations, especially C>T. Our collaborators brought us new data from their previously described cancer transformation studies [30]. Briefly, YAMC cells are transformed by exposure to polarized macrophages (M φ) or 4-hydroxy-2-nonenol (4-HNE). This meant we had mutations from the exposures proper which might further be clonally expanded by the culture model — greatly increasing our ability to detect somatic mutations.

**Fig. 2:**
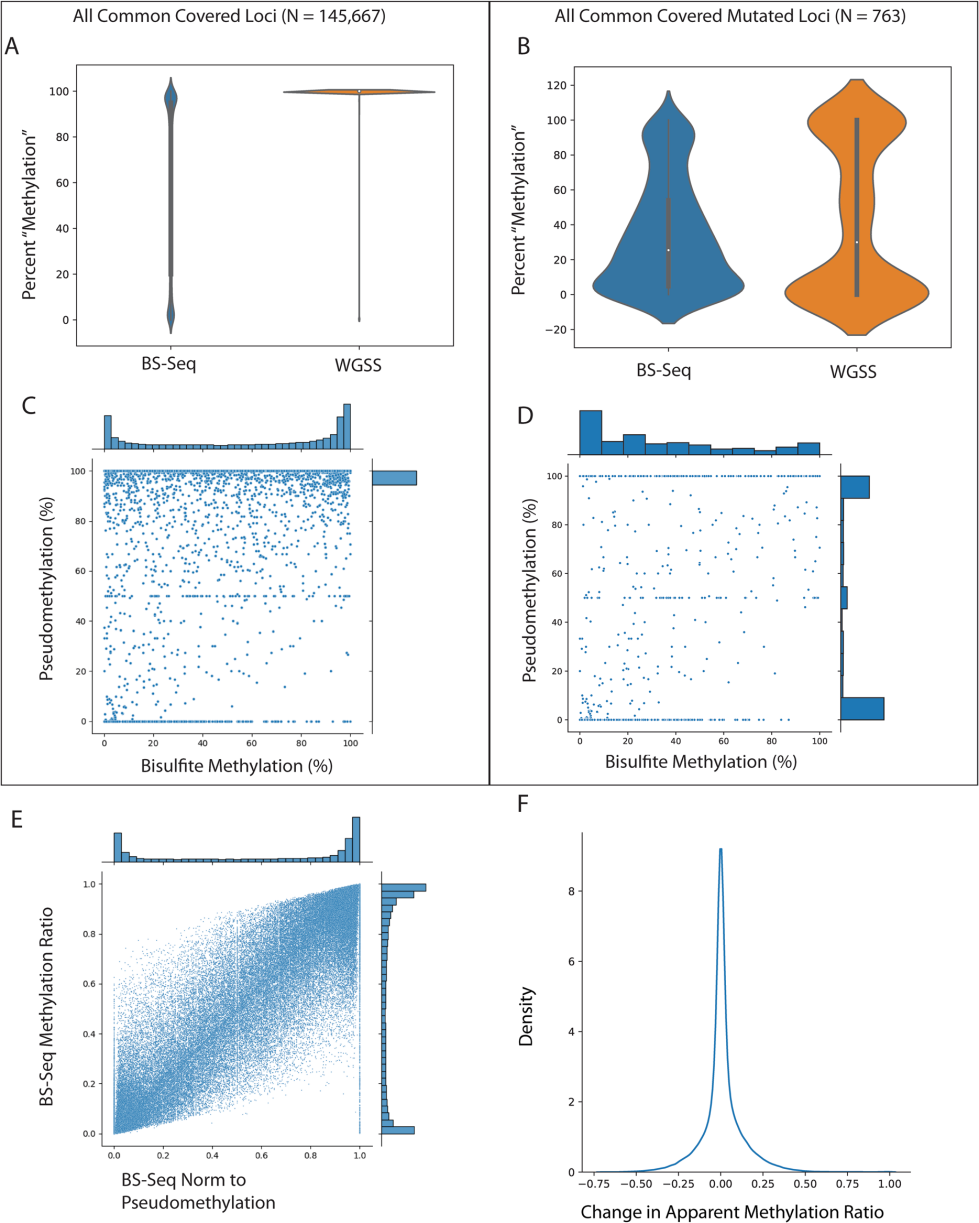
Inducing somatic mutations in vitro reveals the influence of mutations on epigenetic readouts. Bisulfite oligonucleotide capture sequencing (BOCS) and whole-genome shotgun sequencing (WGSS) data from cancer clones (*n* = 6) were analyzed using Bowtie2 and Bismark. **A** Overall distribution of methylation (BOCS) and pseudomethylation (WGSS) at all sufficiently covered loci. **B** Distribution of methylation and pseudomethylation in loci with confident mutation calls. **C** Relationship between pseudomethylation and methylation at all loci (Pearson *r* = 0.0914). **D** Relationship between pseudomethylation and methylation at confident mutation-called loci (Pearson *r* = 0.5750). **E** Relationship between standard bisulfite and bisulfite sequencing adjusted for pseudomethylation level (methylation/pseudomethylation, M/P) at each locus. **F** Distribution of changes in apparent methylation (methylation — (methylation/pseudomethylation)) across all loci

We performed high-coverage whole-genome shotgun sequencing (WGSS, average coverage 30 ×) to detect mutations and BOCS (average coverage over targets 15 ×) to detect DNA methylation. In addition to analyzing the WGSS data for variants, we processed the “unconverted” reads using the same tool we used for calling methylation from the BOCS libraries [37]. This let us generate “pseudomethylation” measurements, or the apparent methylation state of raw DNA. These together allowed us to explore the relationship between DNA methylation and somatic variants (Fig. 2). The BOCS-derived methylation level showed the expected bimodal distribution (Fig. 2A). Meanwhile, the WGSS-derived pseudomethylation exhibited a Zipfian distribution with a mode at 100%. This is consistent with most loci matching the reference genome, and the tail being created by mixed C/T reads induced by mutations. In addition, we observed a negative correlation between methylation level and variant allele frequency (Pearson* r* = − 0.2597). Since these data were true pairs (aliquots of DNA from the same samples), we compared the bisulfite methylation levels to both pseudomethylation and independently performed variant calls (“Methods,” Supplemental Table 1). We restricted the analysis to loci with greater than 10 × coverage in the bisulfite-tolerant alignment performed by Bismark in both the BOCS and WGSS datasets, leaving us with 145,667 loci detected in both unconverted and converted sequencing data across all samples (Fig. 2C). We further restrict some analyses of these common sites with independently called variants by intersecting the loci covered in the methylation readouts with SNVs/indels using bedtools, leaving 763 confidently measured methyl/pseudomethyl/variant triples. These loci with confident variants exhibited both a reduction in their bisulfite-determined methylation level, as well as a distribution reminiscent of the overall BOCS distributions generated by unconverted WGSS sequences (Fig. 2B). We observed that the correlation between methylation signal and “pseudomethylation” from WGSS was low (Pearson *r* = 0.0914) in the overall data (Fig. 2C). The correlation is further depleted when removing any loci with mutations (Pearson *r* = 0.0400), and greatly enriched in mutation-adjacent loci (Pearson *r* = 0.5750) (Fig. 2D). We then attempted to correct for the loss of methylatable cytosines occurring at the mutated loci. To do this, we divided each locus’ methylation level by its pseudomethylation level, generating an M/P ratio. Interestingly, loci across the distribution of observed methylation levels were completely explained by pseudomethylation, as exhibited by sites with M/P of 100 despite lower percentages observed in BOCS reads (Fig. 2E). While we originally hypothesized that C>T mutations would simply drive methylation estimates down, we also observe many loci with 0 M/P despite observing non-zero methylation levels (Fig. 2E). In order for this value to occur, the denominator pseudomethylation value must be near zero (implying near complete C>T conversion compared to reference) while the bisulfite-seq observes some proportion of methylated reads. We believe this effect is driven by the difference in selection strategy, since the whole-genome reads are derived from less-biased shotgun sequencing, while the bisulfite data relies upon capture probes which may bias toward methylated reads to survive capture selection, or alternatively is driven by the known bias of bisulfite-based sequencing which can enrich libraries for methylated regions [41]. This may reflect an observational bias toward unmutated sequences, due to either capture probe stereochemistry/kinetics or by methylated reads being more likely to survive the harsh bisulfite treatment. While these results were exciting for our proof of concept experiment, their extent and frequency must be understood. Even in an in vitro system including induced mutagenesis, most of the loci are unaffected by mutation readouts (Fig. 2F). However, some loci have their apparent methylation level misestimated by 50 + %. These results suggest that mutations do in fact influence methylation readouts, and it may be possible to deconvolve methylation signals with paired mutation distributions.

### Epigenetic clocks can be trained on microarray data without bisulfite conversion of DNA

Given our demonstration that it was possible for genetic information to leak into methylation readouts in sequencing data, we were curious if the changes driving pseudomethylation levels in sequencing contexts also affect methylation microarrays and, subsequently, epigenetic clocks. We hypothesized that chemical alterations to DNA other than methylation may occur during aging, other time-related processes [42], or induced during sample prep, that could be detected unintentionally by methylation arrays and contribute to age predictions. To this end, we generated two datasets from 38 samples split for parallel processing. The first dataset was processed as indicated by Illumina [13], generating apparent methylation data from bisulfite-converted DNA samples (converted), while the second was generated using the same workflow but without the addition of sodium bisulfite (unconverted). These datasets were then used to train and evaluate age-predictive models and explore their respective information content. In order to evaluate not just our bespoke dataset, but human epigenetic clocks and the properties of unconverted data at different scales, we approached this problem from multiple directions. We analyzed the age-predictive loci from our converted and unconverted clocks, and by published human clocks, to identify commonalities that may help us understand if human clocks are affected. Finally, we tested the ability of human genotyping array data to directly predict age by training “clocks” on both allele calls and raw array signal.

**Fig. 3:**
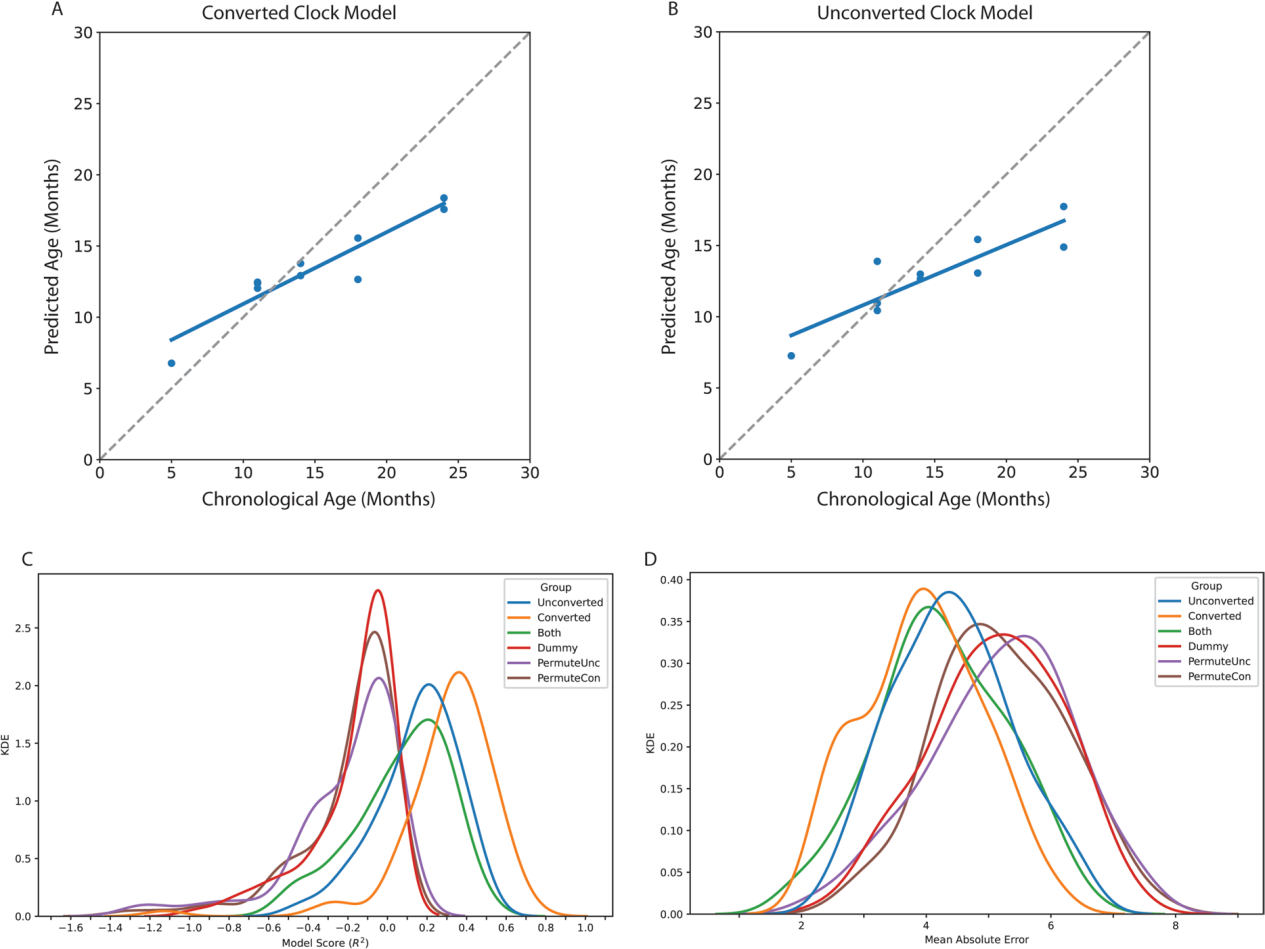
Epigenetic clocks can be trained on mouse methylation microarray data with or without bisulfite conversion. DNA was isolated from dissected mouse hippocampi throughout the mouse lifespan (*n* = 38). In parallel, DNA was processed with standard bisulfite conversion (converted) or a mock conversion process lacking solely the bisulfite reagent (unconverted) for each of the 38 samples. Models were trained on converted and unconverted data. Example fits are shown for one iteration’s test set for converted (**A**) and unconverted (**B**) data. To obtain an estimate of age prediction accuracies, 75/25 train/test splits were repeated for 100 iterations to obtain distributions of prediction accuracy (**C**) and error (**D**). The distribution of observed model scores and errors were compared using one-way ANOVA and Tukey’s post hoc tests with two-tailed significance testing. Models trained on converted, unconverted, and both datasets combined were all significantly better than a dummy model predicting the mean age, as well as models trained on samples with permuted ages. The converted models were also significantly better than unconverted alone or both

Since we could not locate public datasets using unconverted DNA on methylation arrays, and to leverage the paired dataset we have generated, we took a two-layered cross-validation approach. In addition to cross-validation within training data during the regression, we iteratively split our data into 75/25 train/test splits. We then generated 100 clocks for each resampling of both converted and unconverted datasets (Fig. 3) to compare how often the models can predict age at all, their relative accuracies, and how the loci chosen affect these outcomes. Significant main effects were detected for both model score (*F*(5, 595) = 77.58*, p* = 1.4 × 10^−62^) and model error (*F*(5,595) = 41.49, *p* = 1.2 × 10^−36^). We first observed performance significantly better than the dummy or permuted groups from both converted (Fig. 3A) and unconverted (Fig. 3B) models trained on the same train/test split (full significance testing in supplemental Table 2). The models trained on unconverted loci had significantly lower accuracy and higher error than the real methylation data, and training models with both converted and unconverted features did not significantly increase performance, implying redundancy in information. These findings were recapitulated even without primary feature selection (Supplementary Fig. 4) for both score (*F*(2,297) = 86.66, *p* = 3.06 × 10^−29^) and error (*F*(2,297) = 38.90, *p* = 2.06 × 10^−15^). Taken together, these results show we can generate “epigenetic” clocks even without the application of sodium bisulfite which encodes the methylation status into a discernable base difference. However, it is still undetermined if the methylation array signal detected with unconverted DNA is directly caused by DNA mutations or rather influenced by local changes to the genome and epigenome.

### Understanding the relationship between converted and unconverted epigenetic clocks

**Fig. 4:**
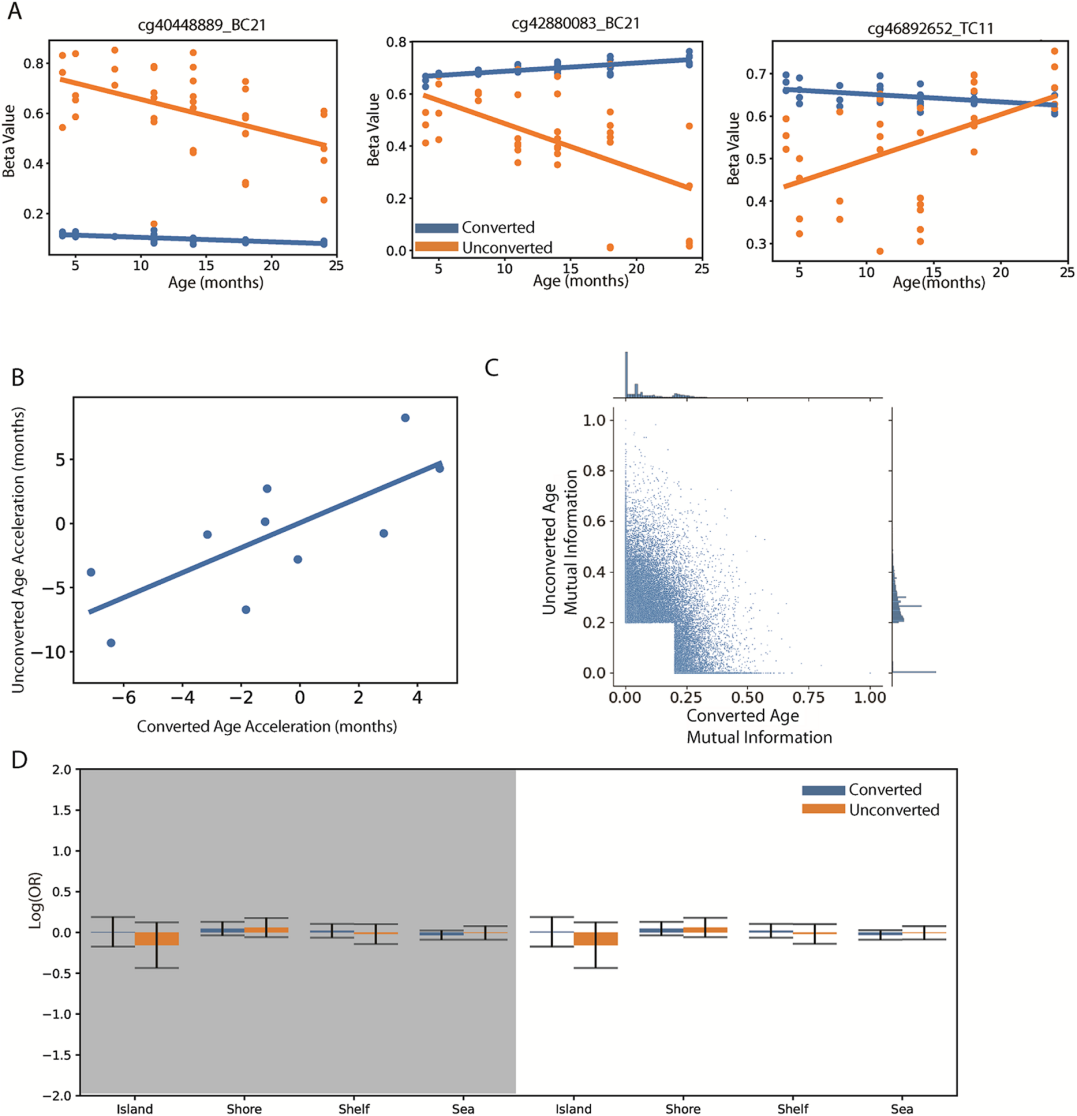
Converted and unconverted DNA are predominantly age predictive due to unique signals but share genetic features and overall trends. **A** Three example loci that were age predictive in both data types. Lines show linear fit for unconverted (orange) and converted (blue). All common loci’s correlation to age can be seen in supplemental Fig. 1. **B** Comparative age acceleration on the test set of converted and unconverted clocks shows a positive association of age acceleration (Pearson *r* = 0.5713, *p* = 0.0002). **C** Univariate analysis shows loci that would pass primary feature selection are largely independent, but some fraction share information. **D** Genomic window analysis shows no over/underrepresentation of clock sites in either data type. Error bars show the 95% confidence interval for the odds ratio estimates

The observation that unconverted DNA could produce comparable epigenetic clocks led us to question if the clocks were detecting common signals shared between the converted and unconverted datasets, or if the underlying molecular changes were unique to each data type (Fig. 4). To explore this, we analyzed common age-predictive sites of each data type with age (Fig. 4C), the direction of predicted “age acceleration” within the same train/test split (Fig. 4B), as well as their data distribution (Fig. 4A) and genomic distribution (Fig. 4D). We observed that most of the loci selected by one data type were not age predictive in the other by multiple metrics. First, we found that sites with high mutual information (MI) in either converted or unconverted were largely unique — having a large peak at 0 MI across data types (Fig. 4C). Some loci exhibited comparable MI in both data types and are likely candidates for whatever information explains unconverted clocks leaking into epigenetic data. The sites that appeared in at least one clock from either modality were close to randomly expected overlap (representation factor 0.98, *p* = 0.679), again implying that the unconverted and converted models are neither biased toward nor against each other. The correlations of the commonly selected loci with age were generally positive in the converted and negative in the unconverted, but all combinations were observable (Suppl. Fig. 1). The absolute differences in apparent “methylation” and their variability were noticeably larger in the unconverted samples (Fig. 4A). Despite their seemingly unique age-predictive information, there was not an obvious difference in their biological characteristics. There was no statistical difference in their abundance across CpG islands or coding features (Fig. 4D). Despite the orthogonality of their underlying data, there was a significant positive correlation between age acceleration across modalities (Fig. 4B).

In summary, these findings provide evidence that (1) age-predictive DNA methylation changes are largely independent from the unconverted aging signal, but as many as 5% of loci on the mouse methylation array are age predictive with and without bisulfite conversion; (2) there is no obvious genomic region/sequence feature to enable us to exclude confounded loci a priori; and (3) the use of unconverted DNA signals as age predictors warrants further exploration due to their potential for a unique, orthogonal signal. However, these results are limited to our mouse aging dataset, and it was unclear if human epigenetic clocks might be gaining mutation-derived information. To this end, we explored the relationship between newly annotated regions of allele-specific methylation [28] and multiple human epigenetic clocks, as well as the potential of mutations alone to provide direct age prediction using public human data.

**Fig. 5:**
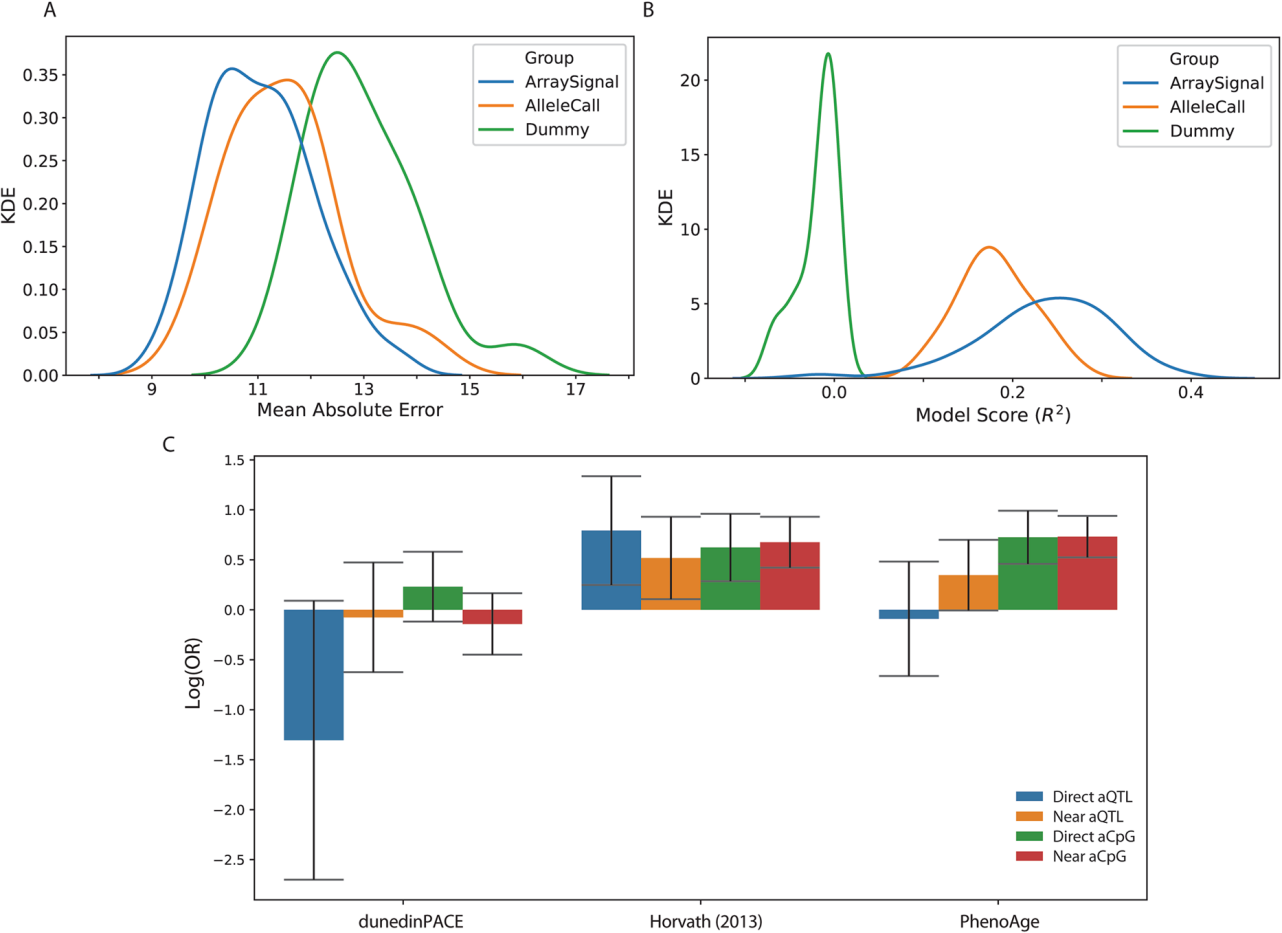
Mutations are weak age predictors alone, but downstream effects on methylation are overrepresented in human epigenetic clocks. A public dataset of human genotyping array data was used to construct clocks using trinary allele calls (AlleleCall) or fluorescence intensity directly (ArraySignal) (*n* = 203). Both were significantly more accurate (**A**) and had significantly lower MAE (**B**) than the dummy model despite poorer performance than converted or unconverted methylation array data. Distributions of observed scores and error were compared using a one-way ANOVA with Tukey’s post hocs for between group differences and using two-tailed significance testing. **C** Comparison of locations with allele-specific methylation relationship to human epigenetic clocks [28]. Loci were split into directly mutated positions (aQTL) and the CpGs they impinge upon (aCpG), as well as direct overlap and nearby (within 25 bp). Horvath’s first chronological clock was overrepresented in both aQTLs and aCpGs, while PhenoAge was enriched in only the affected CpGs. Meanwhile, loci from the longitudinal model DunedinPACE were biased against mutation-derived information but not depleted in loci downstream of mutational effects. Error bars show the 95% confidence interval of the odds ratio estimates

While it is known that germline mutations cast a discernable shadow onto epigenetic data by direct deamination [18], an exploration of the more subtle effects that local genomic sequence has on epigenetic modifications has been occurring outside of the epigenetic clock discussion [28]. Stefansson et al. identified quantitative trait loci (QTLs) which predicted the methylation level of proximal cytosines in an allele-specific manner (termed ASM-QTLs) in a cohort of > 7000 human blood samples. We compared the distribution of both QTLs underlying allele-skewed methylation phenomena (aQTLs), and the CpGs whose methylation these impinge upon (aCpGs) to multiple epigenetic clocks (Fig. 5C). When compared to their respective backgrounds, the chronological age predictor from Horvath (2013) [3] showed a significant overrepresentation in both aQTLs and aCpGs, implying the model could be gaining information from the overlapping QTL, as well as downstream through *bona fide* methylation differences they cause. PhenoAge [4], however, was neither over nor underrepresented in QTLs directly; it was enriched in loci whose methylation difference is best explained by a sequence variant. Finally, the DunedinPACE [39] clock was the most robust to these aQTLs, but still did not select against aQTLs nor aCpGs. Taken together, these findings imply that epigenetic clocks at worst are gaining direct information from genotype, and at best failing to discern between loci with real epigenetic changes and loci with other genomic alterations. Furthering this, we analyzed 256 samples from another study on allele-specific methylation patterns, which instead collected genotyping data using microarrays [27]. We again used elastic net regression to train aging clocks using both mutation calls (allele) and the fluorescent signal (signal) and found evidence they were significantly better predictors than a dummy model (Fig. 5A and B) by both score (*F*(2,297) = 126.94, *p* = 6.45 × 10^−32^) and error (*F*(2,297) = 30.08, *p* = 1.48 × 10^−11^) (see Supplemental Table 2 for full post hoc test results). However, their performance was markedly worse than epigenetic and unconverted clocks. These data imply that mutations alone do not fully explain the predictive power of either real epigenetic clocks nor our unconverted experiment.

## Discussion

Our first set of experiments provided support for the hypothesis that somatic mutations can influence estimated DNA methylation levels in bisulfite-conversion assays. Effects of germline mutations have been previously described in the context of array data [18], but somatic mutations are more rarely studied alongside methylation — perhaps due to the unclear nature of their relationship [19]. In human sequencing data, it was found that methylated CpGs have fewer single nucleotide polymorphisms (SNPs) than unmethylated loci. In fact, loci with intermediate methylation levels (20–60%) had the highest mutation rates, but only outside of promoters and CpG Islands [29]. We believe this relationship between mutation rates and methylation level is driven by a mixture of effects which are difficult to disentangle, but which all lead to shifts in methylation readouts. The mechanism we explore directly allowed us to both corroborate prior evidence that germline variants can be detected by methylation data [43, 44], and newly demonstrate that variants outside of allelic frequencies both influence methylation data and, in some cases, can completely explain apparently unmethylated reads (Fig. 2E). Meanwhile, some loci with 0% pseudomethylation had cytosine-containing reads in their bisulfite-sequenced counterpart — implying some portion of reads had methylatable cytosines from somatic variability or the genomic thymines are converted into cytosines during bisulfite treatment. This leads to many other mechanisms that must be explored, such as clonal expansion of some cells with advantageous mutations/epigenetic states. One weakness of our approach was the relatively small portion of the genome we could analyze with high confidence due to our strict coverage thresholds applied across all samples analyzed (Fig. 2). Further studies are necessary to catalogue this relationship across broader genomic regions to maintain the necessary coverage and could ideally assay more than just DNA methylation.

It is also possible that other mutations, such as C>A induced by 8-oxoguanine, inflate the apparent mutation rate [45]. The possibility of 8-oxoguanine base pairing with cytosine or adenine is also parsimonious with the observed data, and with the possibility to alternate across DNA molecules within individual donors. 8-Oxo-dG can also interfere with DNA methylation readouts and are also associated with C>A mutations. In our prior report, we used motif analysis to identify DNA binding sequences near epigenetic clock sites in humans [25]. We saw two families of hits: (1) TEADs and snRNPs — making an interesting link with gene expression, and (2) hnRNPs — proteins associated with telomeric repeats and maintaining DNA secondary structures [46]. Another link that ties epigenetic modifications and 8-oxo-dG is found in alternative DNA structures. Recent reports found that age can be predicted directly from images of nuclear DNA [47], lending some credence to this idea. This has potentially interesting ramifications in epigenetic clock studies, as these structural changes may be the upstream driver of age-predictive methylation changes — allowing somatic mutations, DNA adducts, and other forms of alternative structures to all contribute to a confounded “methylation” readout. Disentangling these variables in future aging studies should allow us to model which sites are *bona fide* epigenetic changes and which are other alterations.

Prior reports found that intermediate methylation values are the most likely to mutate [29]; while we would initially expect that the higher methylation would lead to more problematic deaminations of mC to T [14], these figures do not account for the possibility that mutations directly affected the methylation readout as we observed (Fig. 2). We expect that uracil-DNA glycosylases (UDGs) [48] would lead to increased rates of repair at cytosine-to-uracil mutations, but T:G mismatches could be more error-prone. Enzymes such as thymine DNA glycosylase (TDG) and methyl binding domain 4 (MBD4) repair the T:G mismatches caused by mC>T mutations [49]. However, there is a conserved reduction in thymine excision activity compared to uracil excision activity at T:G and U:G mismatches, respectively. The conserved impairment of glycosylase activity is thought to protect against aberrant base excision at A:T base pairs which are abundant throughout the genome, whereas dU is rare and typically a byproduct of mutagenic DNA damage. Therefore, the activity of these enzymes does not fully protect against C>T mutations, as evidenced by the prevalence of C>T mutations in cancer [50] and aging [51]. The relationship between somatic mutations and age has been explored in the cancer field [52], and the relationship is also dependent on TP53 mutations [53]. The idea that these mutations affect methylation readouts is also discussed, but we could not find direct evidence that somatic mutations could cause apparent loss of methylation. This has potentially interesting ramifications in epigenetic clock studies, as the potential for mutations to influence methylation data, both directly and indirectly, means that clocks may contain information from both mutations and methylation which are currently undetermined. We hypothesize that local DNA structure is upstream of both converted and unconverted age predictions, as is parsimonious with results from ImAge [47], and may make spatial modeling of DNA structure with aging a more interesting target.

Improving our understanding of the mechanisms behind epigenetic clock formation will depend upon our ability to disentangle methylation changes from genetic penumbra in the aging context. We see two ways forward in this regard: specially designed short-read sequencing approaches like double-stranded bisulfite sequencing (DSBS) [54] or five-letter seq [55] would enable us to isolate these two variables by assaying base pairs in a single DNA molecule instead of relying on the reference genome alone. This approach, after bisulfite conversion, will lead to T:G pairs at unmodified cytosines, and A:T pairs at mutated bases. However, many of the reagents initially used in the DSBS assay are no longer available. At the time of this publication, we failed to reproduce the approach — mainly due to inefficient generation of the target ligation products and potential structural problems with the hairpin. Our best-case experiment had less than 0.8% of generated libraries containing the hairpin sequence and Illumina adapter when sequenced using Oxford Nanopore (data not shown).

Another approach is removing the need for bisulfite treatment and post-conversion treatments entirely, which have complex, and perhaps some uncharacterized, effects on DNA bases. Nanopore sequencing could be leveraged to isolate mutations, methylation, and other DNA modifications and by-products of DNA damage simultaneously [56]. There might also be many other modifications present in real DNA samples with aging which influence bisulfite-based assays. Regardless of the approach, it is obvious that bisulfite methods make assumptions that are likely violated in cancer, and the field is actively searching for alternative solutions. The relationship between these same processes and aging will be of great importance to understanding epigenetic clocks and separating the unmodifiable somatic mutations from therapeutically tractable DNA methylation alterations. We are actively collecting nanopore sequencing data throughout the mouse lifespan to enable a richer exploration of the relationship between DNA structure/modifications and aging biology.

Our next set of experiments was targeted at understanding how signals other than methylation may influence the ability of methylation array data to predict chronological age. The approach of mock conversion allows us to capture any variability that is introduced by the other components of the bisulfite preparation but does not allow us to directly measure mutations in general, nor C>T mutations. The data from sequencing reflects the expected results of bisulfite assumptions (Fig. 2), namely that the whole-genome sequencing should report 100% methylation since most reference cytosines are accurate for the samples, but the array data presents another distribution entirely (Supplemental Fig. 2). Nonetheless, these results provide evidence that epigenetic data suffers from being unable to distinguish between real methylation changes and other phenomena that alter the microarray signal. An obvious example is found in the delineation of mC from hydroxymethylcytosine (hmC), where we have previously described how disentangling cytosine modifications can reveal physiological changes otherwise masked by bisulfite preparations [57]. Our results also imply that the relationship between our unconverted data predictions and standard methylation data is unlikely to be due solely to mutations. Our findings provide evidence for both a path to adjusting epigenetic clocks to delineate real methylation changes with age, as well as evidence that some other genomic modification provides an alternative aging biomarker with comparable prediction accuracy.

The approach of using unconverted DNA with methylation arrays comes with numerous limitations. First, the nature of the methylation beadchips involves calculating methylation as a ratio of fluorescence between apparently methylated and unmethylated loci. This hybridization is problematic for the relatively homogenous unconverted DNA samples. Unconverted samples do have notably higher rates of failed probes (~ 55% failing QC), unlike the comparable alignment rates we observed in the sequencing data. Despite this, the unimputed samples have > 100,000 passing probes each, more than four times the number of probes available in Horvath’s clock training set [3], and are capable of training models with age predictions significantly better than chance (Supp Fig. 4). Second, the unconverted DNA does not reflect the mutation-derived pseudomethylation we observed in our sequencing data (Fig. 2). We believe the strange distributions observed in unconverted data (Supp Fig. 2) are artifacts of the ratio-driven approach and preprocessing steps for background and dye-bias correction. The absolute intensity of images from unconverted samples were orders of magnitude lower in both channels for type II probes, but not in type I probes. After the rescaling done during Noob normalization, the observed values are compressed in type I probes and approach the mean value in the extension-based type II probes (Supp Fig. 2). Despite this, there was no bias toward either probe type in selected age-predictive sites, implying whatever makes unconverted DNA predictive is robust to the normalization creating two different distributions. Third, this approach prevents us from stating that genetic variants explain array-based clock signals. Despite this, the ability to predict age with unconverted samples warrants further study. If this finding is robust across multiple datasets, it would imply that bisulfite arrays capture more information than just methylation state. We observed in a reasonable cohort of human data that genotyping arrays could not generate the same level of age prediction as our unconverted dataset (Fig. 5A, B), implying unconverted age predictions are NOT driven by genomic variants directly. However, we show that human epigenetic clocks are likely gaining information about variants directly and indirectly (Fig. 5C). We believe a tripartite relationship between genomic variants, physical DNA structure, and real methylation alterations is combined into an aggregate signal by bisulfite-based methods which are exploited to predict age, as evidenced by cross-sectional clocks’ bias toward aQTLs and/or their affected CpG loci (Fig. 5C).

### Conclusion

Our data leads us to believe that bisulfite studies of DNA methylation, in both sequencing and array contexts, contain additional information from other genomic alterations. In addition to the known influence of germline variants, we show that somatic variants induced by mutagenic stimuli in vitro covary with apparent losses of methylation. Further, we show loci where non-germline variants are competing with methylation signals, leading to both apparent losses at sites of C>T mutations, as well as loci with bi-allelic germline C>T variants having apparently methylated reads. We further demonstrate that unconverted DNA applied to methylation microarrays in mice and genotyping microarrays in humans can predict age better than naïve or permuted predictors. Finally, we show that popular epigenetic clocks are not free from direct mutation signals, nor loci whose methylation state is largely determined by sequence-level changes. These results provide direction for adjusting future clock studies, as well as focusing epigenetic theories of aging on true epigenetic alterations and avoiding the litany of confounding variables that are currently ignored.

## Supplementary Information

Below is the link to the electronic supplementary material.

Supplementary file1 (DOCX 1.18 MB)
