# Supplementary material for: Methylation array signals are predictive of chronological age without bisulfite conversion
Source: GeroScience. 2025 Jul 29;48(2):2727–42. doi: 10.1007/s11357-025-01785-5 (PMC12972457; doi:10.1007/s11357-025-01785-5)
Supplement: Supplementary file 1 — Supplementary file1 (DOCX 1.18 MB) [file 11357_2025_1785_MOESM1_ESM.docx]

Supplementary Figures

**
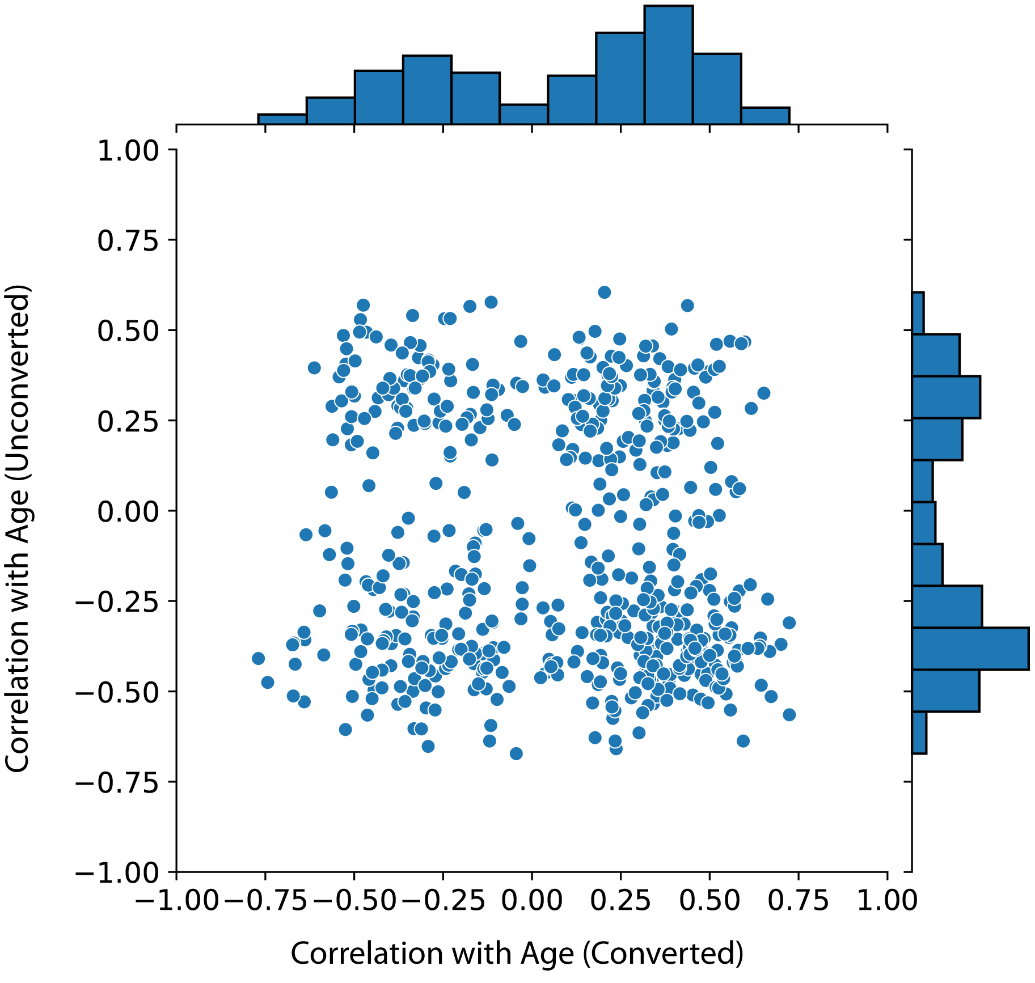
**

**Supplementary Figure 1.** Jointplot of age correlations for each individual locus that was common to at least 1 unconverted and converted clock model. Overall, sites with more positive aging correlations in converted data had lower correlations in unconverted data.

**
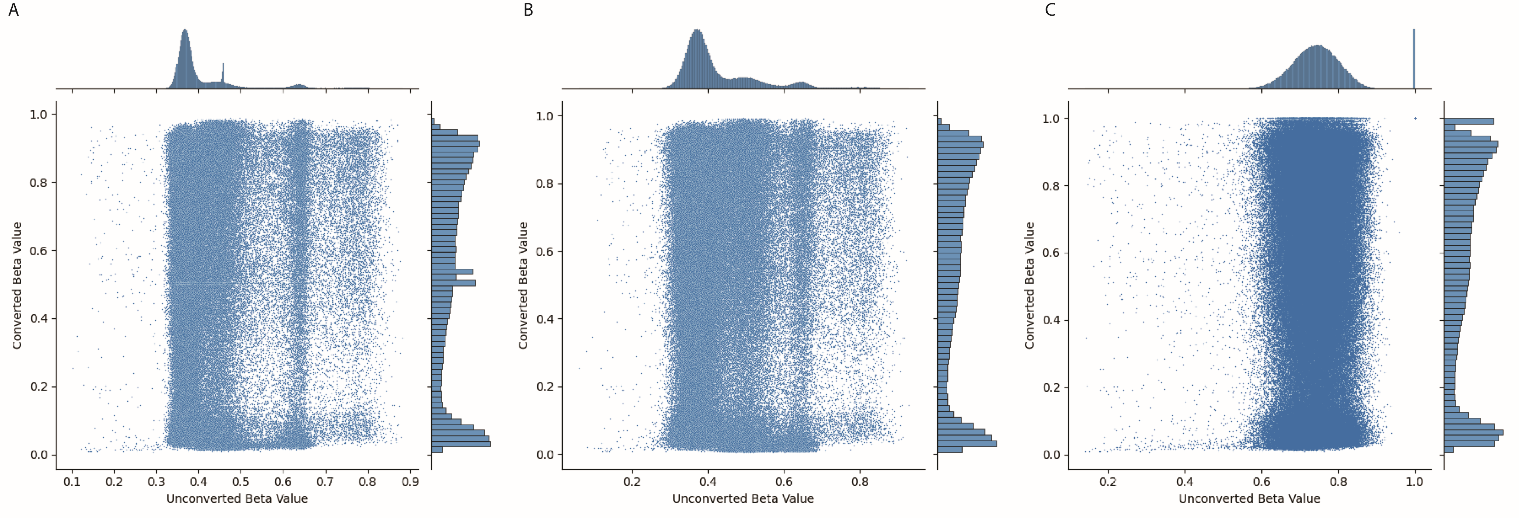
**

**Supplementary Figure 2.** Jointplots of observed distributions of converted (Y) and unconverted (X) beta values. Distribution comparisons are shown for NaNs filled with KNN imputation (**A**), raw data only (**B**) and NaNs filled with 1s to account for potential failure due to lack of diversity in unconverted DNA (**C**).

**
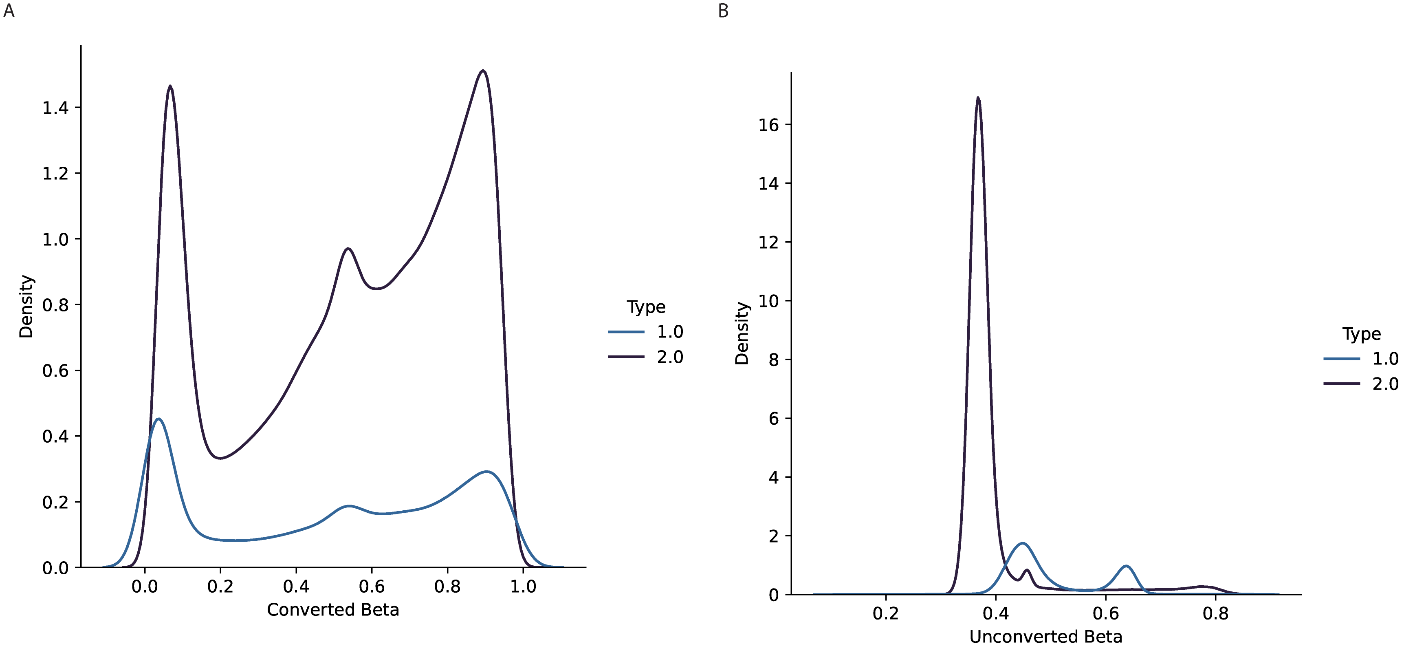
**

**Supplementary Figure 3.** Comparison of mean beta value distributions for Infinium Type I vs Type II probes. Distributions visualized as a kernel density estimate for converted **(A)** and unconverted **(B)** loci.


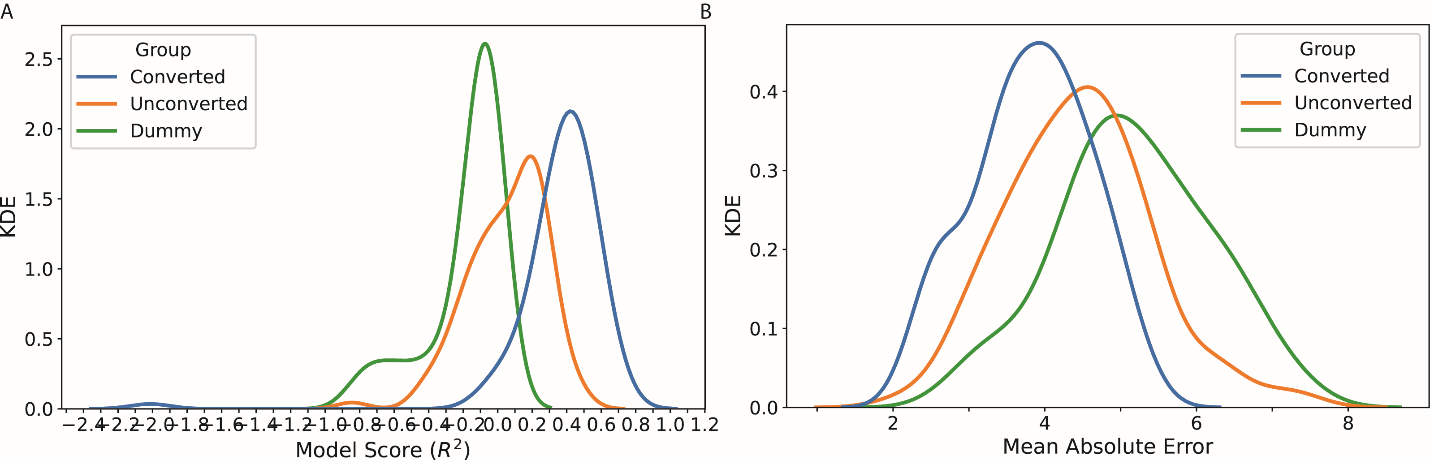


**Supplementary Figure 4.** Elastic net models trained on converted and unconverted DNA without any primary feature selection. Both converted (blue) and unconverted (orange) were significantly better than the dummy model by R^2^ and mean absolute error. Significance was determined using a one-way ANOVA and Tukey’s post-hoc test on 100 iterations of 75/25 train/test splitting as in Figure 3, with two-tailed significance testing. Plots show distribution of R^2^ scores (**A**) and mean absolute error (**B**).
